# Mullerian Duct-Type Clear Cell Adenocarcinoma of the Urethra in a Woman Presenting As Groin Swelling

**DOI:** 10.7759/cureus.67779

**Published:** 2024-08-26

**Authors:** Deerush Kannan, Pratik Taur, Penchala Reddy, Saloni Shah, Narasimhan Ragavan

**Affiliations:** 1 Urology, Apollo Hospitals, Chennai, IND; 2 Pathology, Apollo Hospitals, Chennai, IND

**Keywords:** groin swelling, urethral cancer, mullerian duct, adenocarcinoma, female urethra

## Abstract

Primary Mullerian duct-type clear cell adenocarcinoma of the urethra is a rare clinical entity with a varied clinical presentation. This can be diagnosed only with a high index of suspicion. Clinical examination, biopsy, and immunohistochemistry are essential for diagnosis. Management will need a multimodal approach with a combination of chemotherapy and surgical excision.

## Introduction

Primary malignancies of the female urethra are rare, accounting for <1% of genitourinary malignancies [1]. Primary clear cell adenocarcinoma of the urethra is even rarer in female genitalia. It has a relatively poor prognosis [2,3]. It can develop either from diverticular origin, Mullerian origin, or glandular differentiation of urothelial carcinoma. Knowing the origin of tumor immunohistochemistry is especially useful [4]. We came across one such case that was diagnosed only after the final histopathology arrived, and in view of the disease rarity and the variation in presentation, we describe it in this case report.

## Case presentation

**Figure 1:**
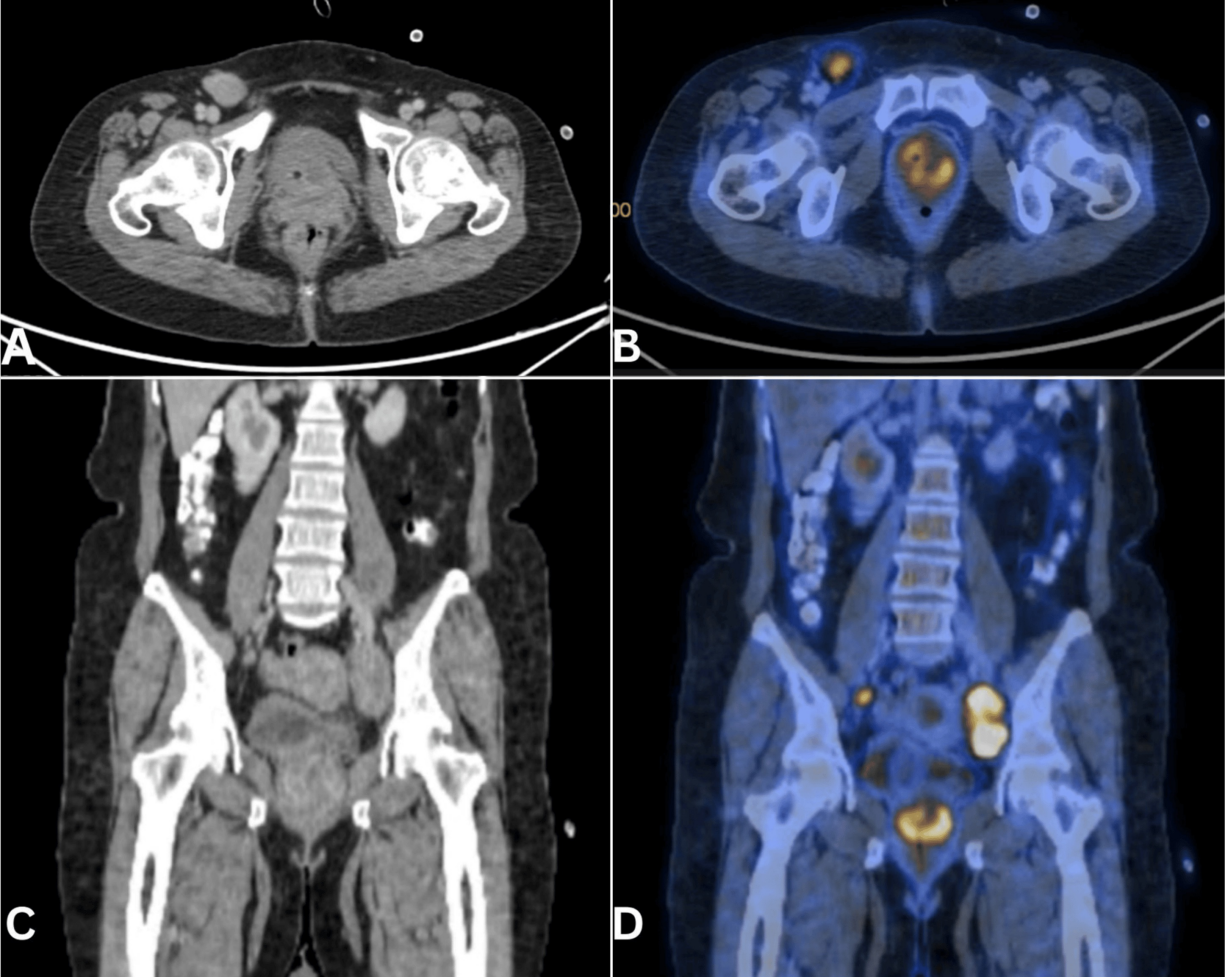
Axial (A) and coronal sections (C) of the CT pelvis with urethral tumor. Axial (B) and coronal (D) sections of PET CT showing metabolic activity CT: computed tomography, PET: positron emission tomography

**Figure 2:**
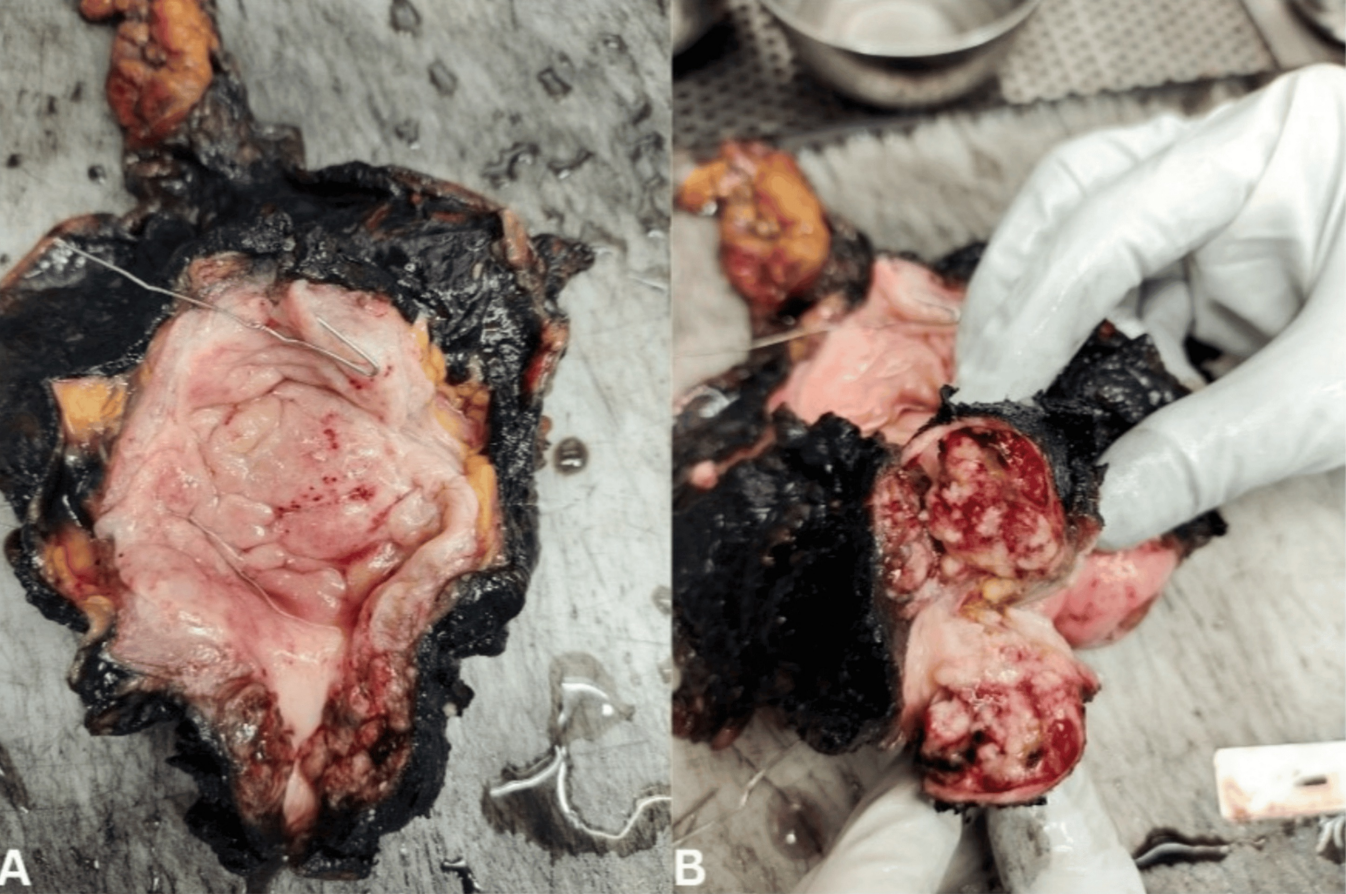
Resected specimen of the bladder with urethral tumor

A 39-year-old lady presented with right groin swelling. Clinical (including pelvic) examination revealed right inguinal adenopathy and palpable urethral mass. Imaging confirmed a urethral mass involving the vagina and right inguinal and pelvic adenopathy (Figure 1). Fine-needle aspiration cytology from the inguinal nodes confirmed adenocarcinoma. Neoadjuvant chemotherapy was given with a substantial reduction in the size of the adenopathy and an improvement in operability. Anterior pelvic exenteration, including a wide margin in the vagina, and pelvic inguinal node dissection were done (Figure 2). Ileal conduit diversion was done. The patient made a good recovery and received adjuvant chemotherapy (three cycles). She is recurrence-free at nine months follow-up. The histology of the resected specimen revealed adenocarcinoma of Mullerian duct origin.

**Figure 3:**
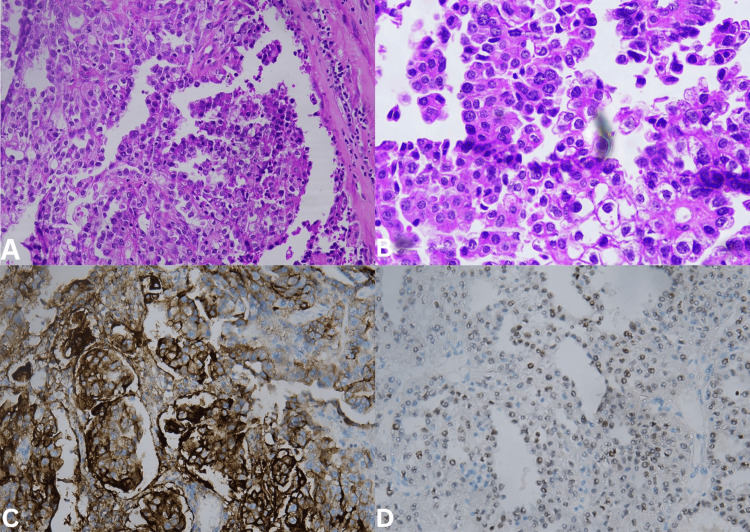
Histopathology hematoxylin and eosin staining image of tumor at 100X magnification (A), at 400X magnification (B), immunohistochemistry staining with Napsin (C), and immunohistochemistry staining with PAX8 (D)

Histopathological examination revealed urethral wall infiltration in muscularis propria by a malignant neoplasm with clear cytoplasm arranged as in the papillary pattern, as sheaths, and with some gland formation with foci of moderate to severe pleomorphism with clearing and hob nailing (Figure 3A-3B), features suggestive of clear cell carcinoma of the urethra. Lympho-vascular invasion was present. Margins were clear, with an uninvolved vaginal wall. One pelvic node and three right inguinal nodes were positive for metastatic carcinoma with treatment-associated changes. Immunohistochemistry was positive for cytokeratin (AE1 + AE3), cytokeratin 7, CA125, Napsin A (Figure 3C), PAX8 (Figure 3D), weakly positive for GATA3, PR, ER, and negative for CK20, P63, and vimentin, consistent with clear cell adenocarcinoma of Mullerian origin.

## Discussion

Primary urethral carcinoma (PUC) is a rare disease, especially in women [5]. Overall, the most common histological type of PUC is urothelial carcinoma (54-65%), followed by squamous cell carcinoma (16-22%) and adenocarcinoma (10-16%) [5]. In women, adenocarcinoma is a more common histology (38-46.7%), followed by squamous cell carcinoma (25.4-28%), urothelial carcinoma (24.9-28%), and other histological entities (6%) as per the Surveillance, Epidemiology, and End Results (SEER) database.

Clear cell adenocarcinoma has a congenital origin [6], is from the paraurethral ducts [7], or is proliferating renal tubular cells in the urinary tract [7]. The clinical presentations of urethral carcinoma are varied. Patients may present with hematuria, bloody urethral discharge, urethral mass, bladder outlet obstruction, pelvic pain, urethrocutaneous fistula, abscess formation, and dyspareunia [8]. Our patient presented with groin swelling. Further examination revealed the urethral mass. The urethra in women drains both into the inguinal and pelvic nodes. Hence, all the drainage basins should be assessed [9].

## Conclusions

Mullerian duct-type clear cell adenocarcinoma of the urethra is exceedingly rare in women. Groin swelling can be the only presenting symptom; hence, assessment of all the drainage areas is essential. For a precise diagnosis or to know the origin of the tumor, immunohistochemistry stains are essential. These tumors should be treated aggressively because of the poor prognosis of the condition. More case studies or large-scale case series are required to better understand the long-term prognosis and treatment options.
